# The Role of Blood Viscosity in Infectious Diseases

**DOI:** 10.7759/cureus.7090

**Published:** 2020-02-24

**Authors:** Gregory D Sloop, Quirijn De Mast, Gheorghe Pop, Joseph J Weidman, John A St. Cyr

**Affiliations:** 1 Pathology, Idaho College of Osteopathic Medicine, Meridian, USA; 2 Internal Medicine, Radboud University Medical Center, Nijmegan, NLD; 3 Cardiology, Radboud University Medical Center, Nijmegen, NLD; 4 Internal Medicine, Independent Researcher, Columbia, USA; 5 Cardiac/Thoracic/Vascular Surgery, Jacqmar, Inc., Minneapolis, USA

**Keywords:** hemorheology, blood viscosity, infection, atherothrombosis, myocardial infarction, acute phase response, atherosclerosis, inflammation, anemia of chronic disease, thrombosis

## Abstract

Blood viscosity is increased by elevated concentrations of acute phase reactants and hypergammaglobulinemia in inflammation. These increase blood viscosity by increasing plasma viscosity and fostering erythrocyte aggregation. Blood viscosity is also increased by decreased erythrocyte deformability, as occurs in malaria. Increased blood viscosity contributes to the association of acute infections with myocardial infarction (MI), venous thrombosis, and venous thromboembolism. It also increases vascular resistance, which decreases tissue perfusion and activates stretch receptors in the left ventricle, thereby initiating the systemic vascular resistance response. This compensates for the increased vascular resistance by vasodilation, lowering hematocrit, and decreasing intravascular volume. This physiological response causes the anemias associated with malaria, chronic inflammation, and other chronic diseases. Since tissue perfusion is inversely proportional to blood viscosity, anemia may be beneficial as it increases tissue perfusion when erythrocyte aggregating factors or erythrocytes with decreased deformability are present in the blood.

## Introduction and background

The role of blood viscosity in pathophysiology has been neglected even though increased blood viscosity affects the presentation and complications of many diseases. Awareness of blood viscosity has provided insight into the anemias of heart failure, inflammation, renal failure, space flight, “hemolytic anemias”, and most notably, the pathogenesis of atherothrombosis [1-3]. These insights should supplement or even supplant the current views of these conditions. The studies have also shown that increased blood viscosity may explain the excess number of cardiovascular deaths associated with dietary saturated and trans fats and the failure of cholesteryl ester transfer protein inhibitors, some of which increase both cardiovascular- and other disease-related mortality despite increasing plasma high-density lipoprotein concentrations [4,5].

This review examines the fundamentals of blood viscosity and explores its role in several topics of interest to infectious disease specialists: the association of infectious diseases and immunization with myocardial infarction (MI) and venous thrombosis, and the anemias associated with malaria and chronic inflammation and disease. In this review, the anemias associated with acute and chronic infections, inflammation, and diseases such as renal failure, congestive heart failure, diabetes, and neoplasia will be collectively referred to as anemias of chronic disease. 

Determinants of blood viscosity

**Figure 1:**
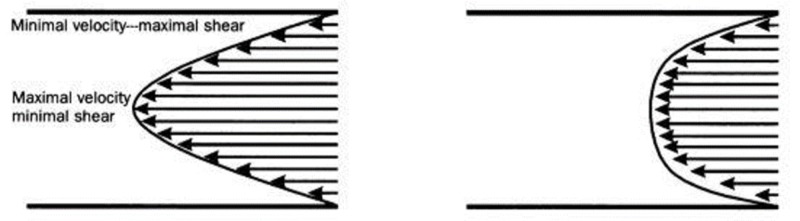
Shear rate Flowing blood can be envisioned as an infinite number of independent layers sliding against each other. In this figure, the velocity of a layer is proportional to the length of an arrow. Shear rate is defined as the difference in velocities between any two layers divided by the distance between them. In a low viscosity fluid (left), the velocity gradient is greater than in a high viscosity fluid (right)

Hematocrit is the most powerful of the variables that determine blood viscosity. Other important variables include plasma viscosity, erythrocyte deformability, and erythrocyte aggregation (rouleaux formation). Importantly, blood viscosity is also determined by its shear rate [6]. A flowing fluid such as blood can be envisioned as an infinite number of parallel layers sliding against each other. Viscosity results from friction between adjacent layers. The shear rate of a fluid is the difference in velocity between any two layers divided by the distance between them. Viscosity is the ratio of shear stress, defined as the force applied to a fluid divided by its contact area, and shear rate (viscosity = shear stress/shear rate). In a low viscosity fluid, a small shear stress (and force) results in a high shear rate (and flow velocity) (Figure 1). 

**Figure 2:**
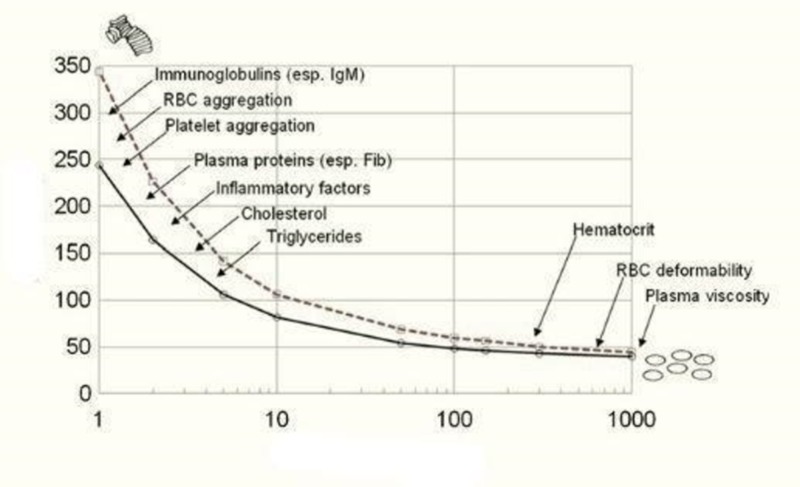
The relationship between blood viscosity and shear rate As shear rate (and velocity) increases from left to right, blood viscosity decreases. Factors that affect blood viscosity are shown in the velocity domain in which their effect is most prominent Figure reprinted courtesy of www.BloodFlowOnline.com

Blood is classified as a “non-Newtonian” fluid because its viscosity varies with its shear rate. In contrast, a Newtonian fluid like water has a constant viscosity at all shear rates. Blood viscosity is minimal at high shear rates and high velocity, as occurs in the systole (Figure 2). This is because erythrocytes reversibly change shape or deform to minimize resistance to flow. At low shear rates and velocities, blood viscosity increases because of reversible, progressive erythrocyte aggregation or rouleaux formation [6]. Low shear rates occur in veins and in arteries in areas of flow separation caused by changing arterial geometry such as dilatations, branches, and curves. Familiar examples of flow separation are the pools and eddies which form when rapidly flowing water encounters a partial obstruction such as a rock.

Erythrocyte deformability is regulated by erythrocyte production of nitric oxide by endothelial-type nitric oxide synthase. The nitrogen donor in nitric oxide synthesis is the amino acid arginine. Thus, erythrocyte deformability and blood viscosity are dependent on plasma arginine concentrations. 

Chemically, blood is a colloidal suspension, meaning it is a mixture of solid particles (erythrocytes, chylomicrons, lipoproteins, etc.) dispersed in another substance, plasma. Erythrocytes are prevented from spontaneously aggregating by their negative surface charge. This attracts clouds of ions that surround erythrocytes and limit how closely they can approach each other. The combination of an erythrocyte’s surface charge and surrounding ions determines its “zeta potential.” The larger the absolute value of the zeta potential, the less likely spontaneous erythrocyte aggregation will occur [7]. 

Any molecule large enough to span the intercellular distance and bind two erythrocytes simultaneously can foster their aggregation. These “bridging” molecules include the acute phase reactants fibrinogen, C-reactive protein, haptoglobin, and ceruloplasmin, as well as Immunoglobulin M (IgM) and low-density lipoprotein (LDL) particles [8]. An example of erythrocyte aggregation caused by bridging molecules is the increased erythrocyte sedimentation rate in patients with hyperfibrinogenemia.

Another process that causes erythrocyte aggregation is the “depletion attraction.” When two erythrocytes approach each other very closely, macromolecules are excluded from the area between them. Contact with macromolecules is limited to the non-apposed erythrocyte surfaces. Brownian movement of the surrounding macromolecules generates a force which drives the two erythrocytes together. The depletion attraction causes the erythrocyte aggregation seen with increased protein concentrations as found in inflammatory and neoplastic hypergammaglobulinemia and dehydration [9].

The pathophysiologic importance of blood viscosity

The Hagen-Poiseuille equation describes flow through a straight tube: Q= ∆P r⁴/8ℓ​η, in which Q = total flow, P = pressure gradient, r = tube radius, ℓ = tube length, and η = viscosity. As noted by Pop et al., increased blood viscosity decreases cardiac output and tissue perfusion [6]. Markedly increased blood viscosity can lead to ischemia and compromise tissue viability, especially in the presence of vasospasm, vascular or pulmonary disease, or marginal blood pressure.

Experimentally, a change in blood viscosity causes a three-fold greater inverse change in blood flow [10]. Thus, blood viscosity is a powerful variable. By decreasing flow, increased blood viscosity increases the risk of thrombosis. Sluggish blood flow is one of the three factors that cause thrombosis, as identified by the German pathologist Rudolph Virchow in the 19th century, the others being hypercoagulability and abnormalities of the vessel wall. Increased blood viscosity creates a prothrombotic state by creating larger areas of slower blood flow (shear rate = shear stress/viscosity). Areas of sluggish blood flow are prone to thrombosis for several reasons. Endothelial production of prostacyclin and nitric oxide, molecules that increase erythrocyte deformability and decrease erythrocyte and platelet aggregability, is shear-dependent [11]. The dispersion of activated coagulation factors and the influx of molecules with fibrinolytic activity are reduced. 

Increased blood viscosity has the potential to cause a vicious cycle: it decreases renal perfusion and oxygen delivery, which upregulates erythropoietin synthesis, further increasing blood viscosity and decreasing renal perfusion, leading to even greater erythropoietin synthesis, etc. Because unremitting positive feedback is incompatible with life, a mechanism to provide negative feedback is necessary to prevent this scenario. In the 1970s, the pioneering hemorheologist Leopold Dintenfass recognized this and proposed that a homeostatic mechanism controls blood viscosity. He thought that many anemias were compensatory to normalize blood viscosity and that malfunction of this mechanism might be a factor in primary or essential hypertension [12]. In 2001, Walter Reinhart also argued that plasma viscosity and hematocrit are under homeostatic control [13]. 

This control is provided by a recently described mechanism called the “systemic vascular resistance response” (SVRR). Increased blood viscosity increases vascular resistance, which is sensed by stretch receptors in the left ventricle. This initiates the SVRR, which normalizes vascular resistance at the cost of anemia. One arm of the response increases the secretion of B-type natriuretic peptide (BNP), which causes vasodilation and natriuresis. Pop et al. have demonstrated that reduction of blood viscosity and afterload by LDL-apheresis reduced the secretion of BNP [1]. The other arm decreases red cell mass by decreasing erythropoietin activity. Acutely, this is accomplished by the expression of soluble erythropoietin receptor, which binds and removes circulating erythropoietin before it can bind to erythrocyte precursors in the bone marrow. The strategy of expressing soluble “decoy receptors” that are unable to transduce a signal is common in the class I cytokine superfamily of receptors, which also includes the receptor to interleukin 6 [2]. In a rat model, increased plasma viscosity decreases renal production of erythropoietin mRNA, which decreases erythropoietin levels over the long term [14]. The resulting vasodilation, natriuresis, and anemia caused by the SVRR decrease vascular resistance. The decrease in red cell mass must be proportional to the decrease in plasma volume to maintain a constant hematocrit. A decrease in plasma volume without a decrease in red cell mass would cause hemoconcentration and increased blood viscosity. Kilbridge, et al. have demonstrated this arm of the response by showing that hyperviscosity blunts the erythropoietin response to hypoxia in mice [2].

In addition to natriuretic factors, the vascular radius is determined by autonomic innervation and numerous other hormones and local factors synthesized by the endothelium. Thus, all modifiable variables in the Hagen-Poiseuille equation are under physiologic control. 

The SVRR has been studied most closely in spaceflight anemia. Upon release from gravity, interstitial fluid from gravity-dependent areas re-enters the circulation. This increases intravascular volume and end-diastolic volume, activating stretch receptors and initiating the SVRR. Plasma volume decreases within hours, followed by decreased erythropoietin concentrations. Upon return to gravity, fluid returns to gravity-dependent spaces. Intravascular volume is restored by the action of antidiuretic hormone, resulting in dilutional anemia until red cell mass is restored [2].

The SVRR is the physiologic antagonist of the renin-angiotensin-aldosterone system (RAAS). The sodium- and volume-conserving and vasoconstrictive activity of the RAAS are well known. Less well known is its erythropoietic activity, which is best seen in posttransplant erythrocytosis. This is defined as a hematocrit >51% or hemoglobin >17 g/dl after renal transplantation; 10-30% of the patients develop thrombotic complications, at least partly attributable to increased blood viscosity. They are treated with angiotensin-converting enzyme inhibitors [2].

The SVRR reacts to increased blood viscosity and vascular resistance during bulk blood flow, which is a characteristic of decreased erythrocyte deformability. The SVRR cannot detect or correct the tendency for thrombosis in areas of flow separation in coronary arteries or in veins.

## Review

The association of infection with myocardial infarction

Acute MI has been associated with bacterial, viral, and parasitic infections. Bacterial infections associated with MI include community-acquired pneumonia, meningitis, pelvic inflammatory disease, staphylococcal septicemia with meningoencephalitis, and gingivitis [15-19]; 7-8% of inpatients with pneumococcal pneumonia develop MI [20]. Thus, MI is a significant complication of severe pneumonia. MI is also noted in association with infections with both bacterial and viral etiologies such as rhinosinusitis and exudative pharyngitis [21,22]. It is also reported in association with influenza and malaria [23,24]. Vaccination for influenza appears to decrease the risk of MI [23].

Increased blood viscosity due to the acute phase reaction is a plausible explanation for these associations. In a prospective study, a one standard deviation increase in blood viscosity raised the relative risk of a cardiovascular event by a factor of 1.2 [95% confidence interval (CI): 1.07-1.36]. This was identical to the relative risk associated with LDL-cholesterol concentration (relative risk = 1.2, 95% CI: 1.07-1.35) [25]. Fibrinogen concentration is also a risk factor for cardiovascular disease. In a meta-analysis, the summary odds ratio for a cardiovascular event in subjects in the upper tertile of fibrinogen concentration compared to the lowest was 2.3 (95% CI: 1.9-2.8) [26].

Several manifestations of infection such as fever, pain-induced tachycardia, and local vasodilation require increased cardiac output and myocardial oxygen demand [27]. The increased vascular resistance and cardiac afterload caused by acute phase reactants further increase myocardial oxygen demand. Hemoconcentration, as caused by decreased fluid intake, increased insensible water losses due to fever, and edema due to increased capillary permeability, increases blood viscosity, afterload, and oxygen demand. These abnormalities could be enough to cause myocardial ischemia and infarction in the presence of pre-existing coronary artery disease or impaired oxygenation due to pneumonia. Increased blood viscosity also increases shear stress on the endothelium, promoting rupture of the thin cap of vulnerable atherosclerotic plaques (shear stress = shear rate x viscosity) [6]. 

Generally, the magnitude of an acute phase response correlates with the severity of an infection. The impact of the acute phase response on blood viscosity explains why the severity of pneumonia correlates with the risk of MI and the increased risk early in infection [20]. Increased blood viscosity associated with severe pneumonia was noted by Sir William Osler in the early 20th century. Before the advent of antibiotics, therapeutic phlebotomy, which decreases blood viscosity, was still routinely practiced by some physicians. Osler wrote: “We employ [therapeutic phlebotomy] much more frequently now than we did a few years ago…. To bleed at the very onset in robust, healthy individuals in whom the disease sets in with great intensity and high fever is good practice. Late in the course marked dilatation of the right heart is the common indication” [28].

As noted above, increased blood viscosity increases the risk of thrombosis. Inflammation also creates a procoagulant state via several other mechanisms, including thrombocytosis and increased concentration of circulating tissue factor. The risk of MI remains elevated for 10 years following severe pneumonia [20]. This is also explained by the increased risk of thrombosis caused by inflammation. Fibrous remodeling or “organization” of a mural thrombus results in a lesion indistinguishable from an atherosclerotic plaque [3]. In a coronary artery, these lesions will increase the long-term risk of MI. 

The incidence of MI in hospitalized malaria patients (n = 1,531) was 1.43% compared to 0.82% in all non-malarial patients (n = 37,368) in one study [24]. Before the development of compensatory anemia, blood viscosity is increased in malaria due to hyperfibrinogenemia, increased erythrocyte aggregation, and decreased erythrocyte deformability [29,30]. Erythrocyte deformability on presentation correlates with the lowest hemoglobin value during the subsequent hospitalization, consistent with the theory that this anemia is a response to normalize blood viscosity (vide infra) [31]. 

*Plasmodium falciparum* produces an arginase resulting in hypoargininemia, decreasing the bioavailability of nitrogen atoms for nitric oxide synthesis by erythrocytes. This decreases the deformability of both parasitized and unparasitized erythrocytes [32]. Malarial infection also causes extensive remodeling of the erythrocyte cell membrane, which decreases its zeta potential, fostering erythrocyte aggregation [33]. These pathological changes are superimposed on microcirculatory obstruction caused by the sequestration of parasitized erythrocytes. 

Because the increased risk of MI associated with acute infection is caused largely by the host immune response, an exaggerated response to active immunization can also increase the risk of MI. Heplisav-B (Dynavax Technologies, Berkeley, CA) is a relatively new hepatitis B vaccine that contains yeast-derived recombinant hepatitis-B surface antigen linked to an oligodeoxynucleotide sequence that binds to toll-like receptor 9. It produces seroprotective antibody in 90-100% of subjects after two vaccinations one month apart, compared to 70.5%-90.2% of subjects receiving three doses over six months of another hepatitis B vaccine, Engerix-B (GlaxoSmithKline Biologicals, Rixensart, Belgium) [34]. A phase-3 trial revealed an imbalance of MI between subjects who received Heplisav-B and Engerix-B. Acute MI occurred in 0.25% of 5,587 subjects who received Heplisav-B vs. 0.04% of 2,781 subjects who received Engerix-B (relative risk = 6.97, 95% CI: 0.92-52.97). A statistical review by the U.S. Food and Drug Administration noted that the optimal way to interpret a CI for safety assessment is that the upper confidence limit is the highest relative risk which can be ruled out for a given sample size. Lesser risks have not been ruled out. For this reason, a post-marketing study to evaluate the occurrence of acute MI in 25,000 subjects is underway. The final report from this study is scheduled to be submitted by June 30, 2021 [35]. 

Koutsaimanis and Rée reported the case of a previously healthy 40-year-old male with no cardiovascular risk factors who was diagnosed with acute MI six days after immunization for cholera [36]. On the day of immunization, he suffered pain, redness, and swelling at the injection site. On days two and three following immunization, he felt feverish and experienced tender, swollen joints. On the fourth and fifth days post-immunization, he suffered two 30-minute episodes of tight retrosternal pain. On the sixth day, the retrosternal pain became continuous and he became increasingly breathless. An electrocardiogram revealed an extensive acute anterior MI. Cardiac enzymes were markedly elevated. This clinical course is consistent with a rising IgM titer and increased blood viscosity due to a primary immune response to vaccination culminating in MI. 

Increased blood viscosity caused by a pronounced immune response is a reasonable explanation for the causal association between vaccination and acute MI, which previously was questioned because no causative mechanism had been identified [37]. An MI caused by a pronounced immune response to vaccination is not inconsistent with the decreased incidence of MI associated with vaccination against influenza. The symptoms associated with the immune response to influenza vaccination are milder than influenza itself, and the symptoms of influenza are generally milder in those who have been vaccinated. 

Testing for blood viscosity is usually available only from reference laboratories. Testing for plasma viscosity is available more widely but is of limited utility as it does not detect the contribution of hematocrit or abnormal erythrocyte aggregability and deformability [38]. Serum viscosity testing is the most useful method for detecting hypergammaglobulinemia and an increased protein concentration due to volume contraction. By far the most valuable laboratory investigation for determining the risk of MI associated with infection is the erythrocyte sedimentation rate (ESR), which is a surrogate marker for the fibrinogen concentration and blood viscosity at low shear rates. An unexpectedly high ESR should alert the clinician to an increased risk of MI. As a final word about the association between MI and infection, a recent review that did not consider blood viscosity concluded that most of these MIs could not be thoroughly explained [20].

Infection and venous thrombosis

Increased blood viscosity is a risk factor for deep vein thrombosis [3,39]. Blood flow is slow in veins; therefore, blood viscosity becomes relatively high (viscosity = shear stress/shear rate). The association between infection and venous thrombosis was reviewed by Tichelaar et al. [40]. They found convincing evidence for a two-fold increase in the risk of venous thrombosis following pneumonia, urinary tract infection, and acute infectious diseases not otherwise specified. They also reviewed a study that showed a decreased prevalence of deep vein thrombosis in subjects vaccinated against influenza. Deep vein thrombosis has also been noted in association with acute osteomyelitis in children [41]. Among chronic infections, active tuberculosis and chronic osteomyelitis have been associated with venous thromboembolism or deep vein thrombosis [42,43]. Immobilization may be a comorbidity that increases the risk of venous thrombosis in acute and chronic infections, particularly chronic osteomyelitis.

Hemolytic anemia caused by malaria

Blood viscosity is increased in malaria on presentation because of hyperfibrinogenemia, increased erythrocyte aggregation, and decreased erythrocyte deformability, as noted above. In a study of hospitalized patients with renal failure due to falciparum malaria, blood viscosity was markedly increased on admission and normalized on recovery because of the SVRR. This was accompanied by a drop in hematocrit of 5.8% [2]. Ninety percent of the decrease in hemoglobin concentration was due to the removal of unparasitized cells. Thus, clearance of erythrocytes is not a response to control the infection. 

Demonstrating the beneficial effect of anemia, a recent review reported that mortality in children and adults with severe falciparum malaria was lowest in those with a hemoglobin concentration between 3.5 g/dL and 5.5 g/dL. In cerebral malaria, mortality was lowest in those with a hemoglobin concentration between 7 and 9 g/dL [44]. The SVRR also causes delayed reticulocytosis in response to anemia due to malaria. 

In the Fluid Expansion as Supportive Therapy (FEAST) trial of fluid bolus therapy in children with severe infections including malaria, mortality was increased in subjects with mild anemia who were transfused compared to those who were not (relative risk = 6.4, 95% confidence interval: 3.1-12.9) [44]. Similarly, transfusion has been shown to either provide no benefit or worsen outcome in hemoglobinopathies and the anemias of chronic renal failure and sepsis [2]. These observations show that anemia can have a survival benefit when there is potential for increased blood viscosity. In addition to increasing hematocrit, transfusion increases blood viscosity because the deformability of packed RBCs is decreased during storage. 

Anemia of chronic disease

Zarychanski and Houston proposed in 2008 that anemia of chronic disease is a beneficial response [45]. Ẑupanić-Krmek et al. reexamined this theory in 2014 [46]. They noted that the treatment of anemia of chronic disease should have a negative effect if it is a homeostatic response. They reported that a meta-analysis of 51 studies of erythropoiesis-stimulating agents in cancer showed higher mortality and risk of venous thromboembolism when the therapeutic goal was a normal hemoglobin concentration compared to a lower one. They also noted that studies of transfusion in critically ill patients (sepsis, congestive heart failure, acute pancreatitis) showed increased mortality with a transfusion threshold of 10 mg/dL vs. 7-8 gm/dL. Finally, a meta-analysis of erythropoietin stimulating agents in chronic renal failure showed excess mortality, arteriovenous shunt thromboses, and poorly controlled hypertension in subjects with a higher target hemoglobin concentration [2]. Venous thromboembolism, arteriovenous shunt thrombosis, and poorly controlled hypertension are all easily attributable to increased blood viscosity. These results support the theory that anemia of chronic disease is a beneficial response as it decreases systemic vascular resistance, increases cardiac output, and improves tissue perfusion.

In contrast, received wisdom suggests that anemia of chronic disease is pathologic and should be treated. Mainstream thought holds that this anemia is due to the “reprogramming” of the bone marrow to produce myeloid cells at the expense of erythroid cells or is a response to sequester iron from invading microorganisms [47]. These rationales are inadequate for several reasons. First, the bone marrow in anemias of chronic inflammation is normal except for increased iron stores [48]. Further, the notion of “reprogramming” does not explain anemias associated with lymphocytic inflammation such as rheumatoid arthritis, lupus dermatitis, chronic transplant rejection, chronic viral illnesses, and noninflammatory conditions such as neoplasia, congestive heart failure, and renal failure. Likewise, the sequestration of iron to prevent its utilization by invading microorganisms is counterproductive in many conditions associated with anemia of chronic disease. It seems unlikely that an organism would retain a response that decreases evolutionary fitness.

These arguments make it clear that anemia of chronic disease is an adaptive, beneficial response caused by the SVRR. Intervention into this condition should be undertaken conservatively and guided by the patient’s blood viscosity data. Increased blood viscosity, which can be caused by abnormal plasma composition and many different erythrocyte abnormalities, is a common, nonspecific abnormality that is easily detected by left ventricular stretch receptors. Normalization of blood viscosity by the SVRR decreases systemic vascular resistance and improves tissue perfusion. Treatment of the anemia of chronic disease with erythropoiesis-stimulating agents or transfusion should be undertaken cautiously and guided by monitoring of blood viscosity.

## Conclusions

Both acute and chronic inflammation has the potential to increase blood viscosity. Because increased blood viscosity increases the risk of thrombosis, a wide variety of infections are associated with MI. Increased blood viscosity decreases tissue perfusion, further increasing the risk of MI, particularly in the presence of pre-existing vascular or lung disease. Because blood is a non-Newtonian fluid, the increase in blood viscosity caused by inflammation is even greater in low-shear conditions in veins, increasing the risk of venous thrombosis. Without negative feedback, increased blood viscosity will cause fatal polycythemia: increased blood viscosity will decrease renal perfusion, which will increase erythropoietin secretion, further increasing viscosity, decreasing perfusion, and so on. The systemic vascular resistance response is the adaptive mechanism that prevents this scenario by decreasing hematocrit and intravascular volume and causing vasodilation. This response causes the anemias associated with malaria and chronic inflammation, thereby reducing blood viscosity and improving tissue perfusion.
